# Psychometrics of patient-reported outcomes measurement information system in von Willebrand disease, inherited platelet function disorders, and rare bleeding disorders

**DOI:** 10.1016/j.rpth.2024.102474

**Published:** 2024-06-13

**Authors:** Evelien S. van Hoorn, Sterre P.E. Willems, Wala Al Arashi, Annick S. de Moor, Calvin B. van Kwawegen, Lorynn Teela, Martijn A.H. Oude Voshaar, Idske C.L. Kremer Hovinga, Roger E.G. Schutgens, Saskia E.M. Schols, Frank W.G. Leebeek, Lotte Haverman, Marjon H. Cnossen, Samantha C. Gouw, Hester F. Lingsma, SYMPHONY consortium and the Dutch research group for PROMIS implementation in inherited bleeding disorders, Marjolein Peters, Michiel Coppens, Marieke J.H.A. Kruip, Lize F.D. van Vulpen, Tessa C.M. van Gastel

**Affiliations:** 1 Department of Public Health, Erasmus MC, University Medical Center Rotterdam, Rotterdam, The Netherlands; 2 Department of Hematology, Radboud university medical center Nijmegen, Hemophilia Treatment Center Nijmegen-Eindhoven-Maastricht, Nijmegen, The Netherlands; 3 Department of Pediatric Hematology, Erasmus MC Sophia Children's Hospital, University Medical Center Rotterdam, Rotterdam, The Netherlands; 4 Center for Benign Hematology, Thrombosis and Hemostasis, Van Creveldkliniek University Medical Center Utrecht, Utrecht University, Utrecht, The Netherlands; 5 Department of Hematology, Erasmus MC, University Medical Center Rotterdam, Rotterdam, The Netherlands; 6 Child and Adolescent Psychiatry & Psychosocial Care, Emma Children’s Hospital, Amsterdam UMC location University of Amsterdam, Amsterdam, The Netherlands; 7 Public Health, Mental Health and Digital Health, Amsterdam UMC location University of Amsterdam, Amsterdam, The Netherlands; 8 Reproduction and Development, Child Development, Amsterdam UMC location University of Amsterdam, Amsterdam, The Netherlands; 9 Pediatric Hematology, Emma Children’s Hospital, Amsterdam UMC location University of Amsterdam, Amsterdam, The Netherlands

**Keywords:** adult, blood platelet disorders, coagulation protein disorders, feasibility studies, patient-reported outcome measures, von Willebrand diseases

## Abstract

**Background:**

Patient-reported outcomes measurement information system (PROMIS) measures can be used to measure patient-reported outcomes. PROMIS measures, including computer adaptive tests (CATs) and short forms, have demonstrated the ability to adequately assess outcomes in patients with hemophilia. It is, however, unclear if PROMIS measures are suitable for patients with von Willebrand disease (VWD), inherited platelet function disorders (IPFDs), and rare bleeding disorders (RBDs).

**Objectives:**

To evaluate the feasibility, measurement properties, and relevance of PROMIS measures in adults with VWD, IPFDs, and RBDs.

**Methods:**

In this cross-sectional multicenter study, adults with VWD, IPFDs, and RBDs completed 9 PROMIS measures and the Short Form-36 version 2 (SF-36v2) electronically. Feasibility was determined by the number of completed items and floor/ceiling effects. Measurement properties included construct validity based on a multitrait–multimethod analysis and reliability using the reliability coefficient and greatest lower bound. Relevance was evaluated based on comparison with the Dutch general population.

**Results:**

In total, 111 patients (median age, 57 years [IQR, 44-67]; 60% VWD, 16% IPFD, 24% RBD) participated. Mean number of items answered varied from 5.3 to 8.7 (range, 4-12) per PROMIS CAT in patients with VWD. Construct validity was supported for all CATs and all instruments had a good reliability (≥0.70). The PROMIS measures had less ceiling effects than the SF-36v2.

**Conclusion:**

The PROMIS measures are a feasible, valid, and reliable alternative for the SF-36v2 in patients with primarily nonsevere forms of VWD. The relevance of the selected measures was limited. Additional research is necessary to evaluate the PROMIS measures in adults with IPFDs and RBDs.

## Essentials

• This study investigated patient-reported outcomes measurement information system (PROMIS).
• In this multicenter study, 111 adults with autosomal inherited bleeding disorders participated.
• All analyzed PROMIS measures are reliable and valid alternatives for the Short Form-36 version 2.
• All studied PROMIS Computer Adaptive Tests can be used in clinical practice.

## 1 Introduction

Inherited bleeding disorders are caused by abnormalities in the hemostatic process and consist of a heterogeneous group of coagulation disorders. Hemophilia A and B and von Willebrand disease (VWD) represent approximately 85% of all inherited bleeding disorders, while inherited platelet function disorders (IPFDs) and rare bleeding disorders (RBDs), including disorders of fibrinolysis, are significantly less prevalent [[1], [2], [3], [4], [5]]. Patients with these inherited bleeding disorders exhibit a wide variety of symptoms and their clinical presentation often overlap [[6], [7], [8], [9], [10]]. Disease severity ranges from asymptomatic or minor bleeding to severe and life-threatening bleeding. In general, patients with mild inherited bleeding disorders frequently present with mucocutaneous bleeding, including easy bruising, epistaxis, and heavy menstrual bleeding [11]. In addition, patients with VWD, IPFDs, and rare coagulation deficiencies may present with persistent bleeding after childbirth, trauma, and/or surgery [12,13]. Patients with fibrinolytic disorders typically present with delayed bleeding following trauma or surgery [14]. Previous research has shown that the bleeding tendency may have a significant impact on a patients’ quality of life [12,15]. Repeated bleeding episodes hamper physical functioning and the ability to perform daily and social activities [12]. Insight into a patients’ health-related quality of life is therefore essential in the management and treatment of all inherited bleeding disorders [16].

Patient-reported outcomes measures (PROMs) are self-reported questionnaires used to gauge patients’ perspectives on their health and well-being as well as the impact of disease and treatment on their lives [17]. PROMs measure one or multiple patient-reported outcomes (PROs) and are often classified as generic (ie, applicable for everyone) or disease-specific (ie., applicable for a certain disease, condition, or treatment) [18]. When used in clinical care, PROMs may improve patient-healthcare professional communication, facilitate shared decision making, and increase healthcare professionals’ awareness of patients’ problems and concerns [19,20].

In the Netherlands, several initiatives have validated and advocated the use of PROs measurement information system (PROMIS) as a tool to measure PROs in patients with inherited bleeding disorders [17,[21], [22], [23], [24]]. PROMIS provides a set of generic, standardized item banks that can be used to evaluate and monitor a broad range of health domains (physical, mental, and social health) in both children and adults [17,22]. PROMIS item banks are based on item response theory (IRT), which enables the application of computer adaptive tests (CATs) [25]. With CAT, after the first question, subsequent questions offered to the patient are based on previous answers [26]. The use of PROMIS CATs therefore reduces questionnaire burden, while providing a more tailored and reliable measurement in comparison to existing PROMs [17,25,[27], [28], [29]]. Previous research has shown that a selection of PROMIS measures, including fixed scales and short forms derived from the item banks, perform well in children and adults with hemophilia [17,21,22]. However, it is unclear if PROMIS measures are suitable for patients with VWD, IPFDs, and RBDs. Due to the X-linked inheritance pattern, the hemophilia population predominantly consists of men. Moreover, hemophilia patients frequently receive prophylactic treatment to prevent (spontaneous) bleeding. The majority of patients with VWD, IPFDs, and RBDs, however, are women and their presentation, diagnosis, and management differ significantly from that of men with hemophilia [9]. This study aims to evaluate the feasibility, measurement properties, and relevance of 9 PROMIS measures for adults with VWD, IPFDs, and RBDs.

## 2 Methods

### 2.1 Study design, participants, and data collection

This cross-sectional study included participants from 3 previously performed nationwide cross-sectional studies on VWD, IPFDs, and RBDs in the Netherlands. These are the Thrombocytopathy in the Netherlands (TiN) study, the Rare Bleeding Disorders in the Netherlands (RBiN) study, and the von Willebrand in the Netherlands—Prospective (Win-Pro) study. The TiN study was performed between 2016 and 2018, and the RBiN study between 2017 and 2019. The WiN-Pro study inclusions were performed from 2019 to 2022. These 3 studies included patients from all 6 hemophilia treatment centers in the Netherlands. The inclusion criteria for the WiN-Pro study were similar to those of the WiN study and the exact inclusion criteria have been published elsewhere [3,9,12,30].

For this specific study, we approached adult Dutch-speaking patients who previously participated in either the WiN-Pro, TiN, or RBiN study and gave permission to be contacted for follow-up studies. The exact inclusion criteria for this study differed per type of bleeding disorder and can be found in the Supplementary Methods. In summary, patients with severe or moderate VWD were included if they participated for 2 years in the WiN-Pro study and received treatment at the hemophilia treatment center Erasmus University Medical Center Rotterdam (Erasmus MC), the Netherlands. Patients from the TiN study were included if they had a confirmed IPFD as defined by the TiN study. Patients from the RBiN study were included if they received treatment at the hemophilia treatment center Nijmegen-Eindhoven-Maastricht, Radboud University Medical Center (Radboud UMC), the Netherlands.

Patients who met the inclusion criteria were invited to participate in this study by email between March 2023 and December 2023. This email included a brief explanation about the study goals, the participant information letter, a link to the study website (https://promis-symphony.nl/) of the KLIK PROM portal, and a personal login code [31]. Participants were required to sign an online informed consent form before they could complete the PROMs. Participants were asked to complete the PROMs within 2 weeks of receiving the invitation email. A reminder to complete the PROMs was sent after 2 weeks. Additionally, participants were contacted by phone if there was no response to the invitation or reminder email for further clarification or if participants requested more information. For this validation study, we aimed to include at least 100 participants as recommended by the Consensus-based standards for the selection of health measurement instruments (COSMIN) guidelines [32].

The Medical Research Ethical Committee of the Radboud University Medical Center (MREC Oost-Nederland) reviewed the study protocol and determined it to be exempt from the Medical Research Involving Human Subjects Act (WMO) (MEC-2022-13847). In addition, this study was included as an amendment of the original Win-Pro and TiN studies. These amendments were reviewed and approved by the Medical Research Ethics Committee of the Erasmus University Medical Center Rotterdam and the Medical Research Ethics Committee of the University Medical Center Utrecht (MREC NedMec), respectively.

### 2.2 Measures

#### 2.2.1 PROMIS measures

In collaboration with the Dutch research group for PROMIS implementation in inherited bleeding disorders, 7 Dutch PROMIS measures were selected and assessed as CAT: v2.0 physical function, v1.1 pain interference, v1.0 fatigue, v1.0 anxiety, v1.0 depression, v2.0 ability to participate in social roles and activities, and v2.0 satisfaction with social roles and activities [23,26,33]. In addition, we assessed the short form v1.1 anger and fixed scale v1.2 global health since there is no CAT version available for these measures. The short form v1.1 anger consists of 5 items and assesses an individual’s self-reported angry mood, negative social cognitions, and efforts to control anger [34]. The fixed scale global health consists of 10 items and evaluates the individual’s health across 5 domains (physical function, pain, fatigue, emotional distress, and social health) as well as the individual’s general health perception [35].

All PROMIS measures use a 5-point Likert scale ranging from 1 (never or not at all) to 5 (almost always, cannot do, or very much), except for the fixed scale global health. Global health uses different response categories for each item (eg, ranging from excellent to poor), includes a visual analog scale from 0 to 10 to assess pain intensity, and produces 2 component scores called physical health and mental health [35]. The CATs automatically stopped when the SE was ≤2.2 (95% reliability) and/or a maximum of 12 items per measure were administered. For the PROMIS CATs, T-scores were obtained using the KLIK PROM portal. The T-scores for the short form anger and fixed scale global health were obtained using the PROMIS Assessment Center Scoring Service (https://www.assessmentcenter.net/ac_scoringservice).

All PROMIS measures use a 7-day recall period, except for physical function, ability to participate in social roles and activities, and satisfaction with roles and activities, which do not use a recall period. For all measures, higher scores represent more of the construct (eg, more anger or better physical function). Dutch thresholds for categorizing the T-scores according to severity of the symptoms are available for all PROMIS measures except the short form v1.1 anger [[35], [36], [37], [38]].

#### 2.2.2 Short Form 36

The Short Form-36 version 2 (SF-36v2) survey is a standardized generic instrument which assesses health-related quality of life across 8 domains: 1) physical functioning, 2) social functioning, 3) role limitations due to physical health problems, 4) role limitations due to emotional problems, 5) general health, 6) mental health, 7) bodily pain, and 8) vitality [39]. The number of items per domain ranges from 2 to 10, and when combined the complete SF-36v2 consists of 36 items. Six of the 8 domains use a 3- to 6-point Likert scale, while the other 2 domains use a yes/no scale. For each domain, scores were converted to a 0-100 scale, with higher values reflecting a better quality of life. The recall period of the SF-36v2 varies from the moment of questionnaire completion to the previous 4 weeks [12].

Additionally, the domain scores can be aggregated into 2 summary scores: a physical component summary and a mental component summary. The component scores were standardized using normative data from the United States general population with a mean score of 50 and an SD of 10 [40].

#### 2.2.3 Patient characteristics

Patient characteristics were collected from the WiN-Pro, TiN, and RBiN studies and included age, sex, type of bleeding disorder, and disease severity. Patients with VWD were categorized as having a severe bleeding disorder if they had von Willebrand factor (VWF) levels (VWF:antigen and/or VWF:collagen binding and/or VWF:activity) ≤10 U/dL and/or factor (F)VIII:C ≤20 U/dL. Patients with VWD and VWF levels between 10 and 30 U/dL and/or FVIII:C between 20 and 40 U/dL were classified as having a nonsevere bleeding disorder. Patients with the IPFDs Glanzmann thrombasthenia and Bernard Soulier Syndrome, and patients with RBDs with a severe deficiency, or with a grade III bleeding were classified as having a severe bleeding disorder [41,42]. Grade III bleeding was defined as spontaneous major bleeding such as intramuscular hematomas requiring hospitalization, hemarthrosis, central nervous system, gastrointestinal, and umbilical cord bleeding [41]. The classification of VWD, IPFDs, and RBDs is consistent with the disease severity classification used in the WiN, TiN, and RBiN study [3,10]. In addition, the use of prophylactic treatment was collected at the moment of PROMs completion. Information on the patients’ race or ethnicity was not collected due to Dutch privacy regulations.

### 2.3 Statistical analysis

All statistical analyses were conducted in R version 2022.07.2+576. Descriptive statistics were used to analyze demographics and clinical characteristics of the participants. All completed questionnaires were included in the analyses.

#### 2.3.1 Feasibility

Feasibility of the PROMIS measures and the SF-36v2 for use in clinical practice was assessed based on the number of completed items (mean [SD], range) and the presence of floor and ceiling effects. For all measures, floor and ceiling effects were defined to be present if more than 15% of the participants reported the lowest or highest possible score [43]. We determined the PROMIS measures to be feasible if the mean number of items and/or the presence of floor/ceiling effects was lower on the PROMIS measures than the SF-36v2.

#### 2.3.2 Construct validity

Construct validity was assessed by examining the Spearman correlations between the PROMIS CATs and the SF-36v2 using a multitrait–multimethod (MTMM) analysis. An MTMM analysis can be used to evaluate the convergent and divergent validity when multiple PROs are measured with ≥2 PROMs. Using the MTMM, we expect to find the highest correlation in the situation when the same PRO is measured (possibly multiple times) with the same PROM, followed by when the same PRO is measured with a different PROM, followed by the situation in which both the PRO and PROM differ. In case the same PRO is not measured multiple times with the same PROM, the reliability coefficient can be used as an estimation of the test-retest correlation (eg, when the same PRO is measured multiple times with the same PROM) [44,45].

In this study, we use the MTMM to evaluate the construct validity of the PROMIS CATs by comparing them to certain SF-36v2 domains. While the PROMIS item banks and SF-36v2 domains do not measure the exact same PRO, the PROsetta Stone project and related previous research have shown that the PROMIS CATs can be linked to certain SF-36v2 domains [46,47]. We therefore assessed the construct validity of the PROMIS CAT physical function, pain interference, fatigue, anxiety, depression, and ability to participate in social roles and activities by comparing them with the SF-36v2 domains physical functioning, bodily pain, vitality, mental health (for both anxiety and depression), and social functioning, respectively.

According to MTMM, construct validity is supported when 1) the correlation between same PRO different PROM (SPDP) (eg, CAT physical function and SF-36v2 physical functioning) is >0.5, 2) the reliability coefficient is larger than the correlation between SPDP, 3) the correlation between SPDP is larger than the correlation between different PRO same PROM (DPSP) (eg, CAT physical function and CAT pain interference), and 4) the correlation between SPDP is larger than the correlation between different PRO different PROM (DPDP) (eg, CAT physical function and SF-36v2 pain interference) [44,45]. If condition 3 or 4 are not met, construct validity can still be supported if a minimum of 75% of the correlations between DPSP or DPDP are smaller than the correlation between SPDP [48].

#### 2.3.3 Reliability

Reliability is the degree to which the measure is free of error. In IRT, on which the PROMIS CATs are based, measurement precision varies across the different levels of the measured constructs which is reflected in the SE. IRT-based SEs can be transformed into classical test theory-based reliability coefficients [49,50]. For example, an SE of 3.2 corresponds to a reliability of 0.90 [26,27,49,51] and an SE of 5.5 corresponds with a reliability of 0.70 [26]. Using the SE of the PROMIS scores, reliability coefficients were calculated for all PROMIS measures [49,52].

The reliability of the SF-36v2 was assessed using the greatest lower bound (GLB) based on a preliminary analysis of the data. The GLB represents the smallest reliability possible given the inter-item correlation between the items of the questionnaire. In skewed data and with unequal inter-item correlations, the GLB has been found to provide more precise estimates of reliability compared to the Cronbach’s alpha [53]. The Cronbach’s alpha is reported in addition to the GLB to promote better reliability estimation practice [54]. Both GLB and Cronbach’s alpha were computed using the “psych” package in R using, respectively, the alpha and glb.algebraic function.

For both the PROMIS measures and the SF-36v2 we assessed its ability to draw reliable conclusions based on the test scores on individual (reliability ≥0.90) and group level (≥0.70) [[54], [55], [56]].

#### 2.3.4 Relevance

The relevance of the PROMIS measures for patients with VWD, IPFDs, and RBDs was determined based on a comparison with the general population. The mean T-scores of the PROMIS CATs were categorized into 4 groups according to the available Dutch thresholds: 1) patients with no limitations or symptoms within normal limits, 2) patients with mild symptoms, 3) patients with moderate symptoms, and 4) patients with severe symptoms [[36], [37], [38]]. The distribution of the Dutch general population across these groups was compared with the distribution of the study participants on the PROMIS CAT physical function, Pain interference, Anxiety, Depression, Ability to participate in social roles and activities, and satisfaction with social roles and activities [36,38]. The distribution of the Dutch general population is unknown for the PROMIS CAT Fatigue and the short form anger. A PROMIS measure was determined to be relevant for patients with VWD, IPFDs, and RBDs if the PROMIS scores negatively deviated from the scores of the general population.

## 3 Results

### 3.1 Patient characteristics

During the study period, 174 patients with VWD, 84 patients with IPFD, and 80 patients with an RBD were approached to participate in this study. In total, 111 patients completed one or multiple PROMs resulting in an overall response rate of 33% (response rate: VWD 39%, IPFDs 21%, and RBDs 33%). Of these 111 patients, 105 completed all PROMs. The median age of the patients was 57 years (IQR, 44-67), and 81 (74%) were female (Table 1). The majority of the patients were diagnosed with VWD (60%), had a nonsevere bleeding disorder (86%), and did not use prophylactic treatment at the moment of inclusion (100%).

**Table 1:** Patient characteristics.

	Overall *N* = 111	von Willebrand disease *N* = 67	Inherited platelet function disorders *N* = 18	Rare bleeding disorders *N* = 26
**Sex**				
Female	81 (73%)	49 (73%)	15 (83%)	17 (65%)
Male	30 (27%)	18 (27%)	3 (17%)	9 (35%)
**Age (y)**				
Median (IQR)	57 (44-67)	59 (46-69)	54 (38-60)	58 (44-66)
**Severity**^a^				
Nonsevere	96 (86%)	61 (91%)	15 (83%)	20 (77%)
Severe	15 (14%)	6 (9%)	3 (17%)	6 (23%)
**Prophylactic treatment**				
Yes	0	0	0	0
No	108 (100%)	67 (100%)	15 (100%)	26 (100%)
Missing	3	0	3	0

VWF:Ag, von Willebrand factor antigen; VWF:Act, von Willebrand factor activity; VWF:CB , von Willebrand factor collagen binding; VWF, von Willebrand factor.

a Patients with von Willebrand factor (VWF) levels (VWF:Ag and/or VWF:CB and/or VWF:Act) ≤10 U/dL and/or factor VIII:C ≤20 U/dL, Glanzmann thrombasthenia, Bernard Soulier Syndrome, and rare bleeding disorders with a grade III bleeding and/or undetectable factor activity were categorized as severe bleeding disorders.

Due to the low response rate and heterogeneity within the patients with IPFDs and RBDs, we present the results of patients with VWD. The results for patients with an IPFD or RBD can be found in the supplementary information (Supplementary Tables S5–S19).

### 3.2 PROMIS measures versus the SF-36v2 in adults with VWD

#### 3.2.1 Feasibility

The mean number of questions answered per CAT varied from 5.3 (range, 4-12) for satisfaction with social roles and activities to 8.7 (range, 5-12) for anxiety, which is higher than the number of questions needed to answer for almost all individual SF-36v2 domains. In total, the mean number of PROMIS CAT items completed was 47 (range, 30–79) and the number of questions completed on the SF-36v2 was 36. (Tables 2 and 3).

**Table 2:** Number of completed items, floor and ceiling effects, and reliability of the PROMIS measures in adults with von Willebrand disease.

PROMIS measure	*N*	No. of items				Floor	Ceiling	Reliability
		Mean	SD	Min	Max	%	%	coefficient
**Computer adaptive tests**								
Physical function	67	6.2	2.8	4	12	0%	0%	0.96
Pain interference	67	7.5	3.8	4	12	39%	2%	0.91
Fatigue	66	6.1	2.8	4	12	3%	0%	0.96
Anxiety	66	8.7	2.4	5	12	3%	0%	0.94
Depression	65	7.2	1.7	4	9	15%	0%	0.93
Ability to participate in social roles and activities	64	6.4	2.9	4	12	0%	11%	0.95
Satisfaction with social roles and activities	64	5.3	2.5	4	12	0%	8%	0.96
**PROMIS short form**								
Anger	63	5	-	5	5	14%	0%	0.90
**PROMIS fixed scale**								
Global health – physical health	62	4	-	4	4	0%	0%	0.82
Global health – mental health	62	4	-	4	4	13%	0%	0.82

Max, maximum; Min, minimum; No., number; PROMIS, patient-reported outcomes measurement information system.

**Table 3:** Number of completed items, floor and ceiling effects, and reliability of the SF-36v2 in adults with von Willebrand disease.

Instruments	*N*	No. of items	Floor	Ceiling	Reliability	
			%	%	Cronbach’s α	Greatest lower bound
**SF-36v2**						
Physical functioning	63	10	6%	21%	0.94	0.98
Role limitations due to physical health problems	63	4	5%	86%	0.88	0.93
Role limitations due to emotional problems	63	3	5%	94%	0.96	0.96
Vitality	63	4	0%	0%	0.82	0.89
Mental health	63	5	0%	0%	0.85	0.92
Social functioning	63	2	2%	40%	0.80	0.80
Bodily pain	63	2	0%	32%	0.91	0.91
General health	63	5	2%	5%	0.87	0.91
Physical component score	63	21	0%	0%	0.83	0.86
Mental component score	63	14	0%	0%	0.77	0.85

SF-36v2, Short Form-36 version 2.

For the PROMIS measures, floor effects were observed in the CATs pain interference and depression (Table 2 and 3). No ceiling effects were observed. Ceiling effects were observed on the SF-36v2 domains of physical functioning, role limitations due to physical health problems, role limitations due to emotional problems, social functioning, and bodily pain. No floor effects were observed in the SF-36v2.

#### 3.2.2 Construct validity

All PROMIS CATs met the criteria for excellent construct validity except for the CATs anxiety and depression (Table 4 and Supplement Table S1). Due to the high correlation between these 2 CATs, the correlation between same PRO different PROM (SPDP) (0.67 and 0.71) was smaller than the maximum correlation between different PRO same PROM (DPSP) (0.74 for both CATs), indicating that these CATs do not meet the third criteria of construct validity according to MTMM.

**Table 4:** Summary of the correlation matrix for construct validity assessment in adults with von Willebrand disease.

PROMIS computer adaptive tests	Same PRO, same PROM	Same PRO, different PROM^a^	Different PRO, same PROM		Different PRO, different PROM	
	Reliability coefficient	Corr. b	Min corr. b	Max corr. b	Min corr. b	Max corr. b
Physical function	0.96	0.92	0.39	0.76	0.35	0.74
Pain interference	0.91	0.92	0.50	0.70	0.22	0.69
Fatigue	0.96	0.86	0.51	0.66	0.23	0.69
Anxiety	0.94	0.67	0.41	0.74	0.09	0.58
Depression	0.93	0.71	0.39	0.74	0.17	0.58
Ability to participate in social roles and activities	0.95	0.67	0.51	0.69	0.30	0.67

CAT, computer adaptive test; corr., correlation; Max, maximum; Min; PRO, patient-reported outcomes; PROM, patient-reported outcomes measures; PROMIS, patient-reported outcomes measurement information system; SF-36v2, Short Form-36 version 2.

a CAT physical function was compared with SF-36v2 physical functioning, CAT pain interference was compared with SF-36v2 bodily pain, CAT fatigue was compared with SF-36v2 vitality, CAT anxiety and depression were compared with SF-36v2 mental health, CAT ability to participate in social roles and activities was compared with SF-36v2 social functioning.

b Absolute correlations.

For both CAT anxiety and CAT depression, 80% of the correlations between different PRO same PROM (DPSP) and different PRO different PROM (DPDP) were smaller than the correlation between same PRO different PROM (SPDP), indicating that both CATs have a good construct validity.

#### 3.2.3 Reliability

All PROMIS measures and SF-36v2 domains had a good group-level reliability (reliability coefficient or GLB > 0.70) (Table 2 and 3). The PROMIS CATs, the PROMIS short form anger, and the SF-36v2 domains physical functioning, role limitation due to physical health problems, role limitations due to emotional problems, mental health, bodily pain, and general health had an excellent reliability on individual level (reliability coefficient or GLB ≥0.90).

#### 3.2.4 Relevance and synthesis of the results

Compared with the Dutch general population, the study population reported slightly lower scores on the CAT physical function, which indicates that the study population experiences more limitations in their physical functioning then the Dutch general population (Table 5). Of the Dutch general population, 32% reported to have mild, moderate, or severe limitations in their physical functioning, while 45% of the study population reported to experience limitations in their physical functioning [38]. On all other PROMIS CATs, the study population reported similar or less symptoms/limitations compared with the general population.

**Table 5:** Distribution of PROMIS CAT scores according to the Dutch thresholds in adults with von Willebrand disease and the Dutch general population.

	Within normal limits	Mild symptoms	Moderate symptoms	Severe symptoms
**Physical function**				
Study population	55%	28%	11%	6%
Dutch general population	68%	13%	17%	2%
**Pain interference**				
Study population	81%	6%	12%	1%
Dutch general population	66%	19%	14%	1%
**Anxiety**				
Study population	76%	17%	6%	1%
Dutch general population	70%	14%	15%	1%
**Depression**				
Study population	72%	19%	6%	3%
Dutch general population	71%	15%	13%	1%
**Ability to participate in social roles and activities**				
Study population	80%	11%	9%	0%
Dutch general population	68%	19%	11%	2%
**Satisfaction with social roles and activities**				
Study population	89%	9%	2%	0%
Dutch general population	73%	11%	12%	4%

CAT, computer adaptive test; PROMIS, patient-reported outcomes measurement information system.

In summary, the mean number of items needed to complete on the selected PROMIS CATs was larger than the SF-36v2. Floor effects were present on 2 PROMIS CATs, while ceiling effects were present on 5 domains of the SF-36v2. The PROMIS CATs Anxiety and Depression had a good construct validity. The other PROMIS CATs had excellent construct validity. All PROMIS measures and the SF-36v2 domains had a good group-level reliability and the PROMIS CATs, short form anger, and the SF-36v2 domains physical functioning, role limitation due to physical health problems, role limitations due to emotional problems, mental health, bodily pain, and general health displayed a good reliability on individual level. Compared to the general population, the relevance of the selected PROMIS CATs was limited (Table 6).

**Table 6:** Synthesis of the results on feasibility, measurement properties, and relevance of the PROMIS measures for adults with von Willebrand disease.

		Feasibility		Construct validity^a^	Reliability	Relevance^b^
PROMIS measure	SF-36v2	Number of items	Floor/ceiling effects			
**CATs**						
Physical function	Physical functioning	+	+	++	++	+
Pain interference	Bodily pain	-	±	++	++	-
Fatigue	Vitality	-	±	++	++	
Anxiety	Mental health	-	±	+	++	-
Depression	Mental health	-	-	+	++	-
Ability to participate in social roles and activities	Social functioning	-	+	++	++	-
Satisfaction with social roles and activities	Social functioning	-	+		++	-
**PROMIS short form**						
Anger					++	
**Fixed scale**						
Global health – physical health	Physical functioning	+	+		+	
	Physical component summary	+	±			
Global health – mental health	Mental health	+	-		+	
	Mental component summary	+	-			

Interpretation: Number of items: + on average the mean number of CAT items shown was lower than the number of items on the SF-36v2, - on average the mean number of CAT items shown was higher than the number of items on the SF-36v2. Floor/ceiling: + no or less floor- and/or ceiling effects then the SF-36v2, ± similar floor- and/ or ceiling effects than the SF-36v2, - more floor- and/or ceiling effects then the SF-36v2. Relevance: + patients reported more symptoms than the general population, – patients reported fewer or similar symptoms than the general population. Construct validity: ++ all construct validity criteria are met, + not all construct validity criteria are met but >70% of the correlations between DPSP and DPDP are smaller than the correlation between SPDP, - construct validity criteria are not met and <70% of the correlations between DPSP and DPDP are smaller than the correlation between SPDP Reliability: ++ reliability coefficient or greatest lower bound ≥ 0.90, + reliability coefficient or greatest lower bound ≥ 0.70, - reliability coefficient or greatest lower bound ≤0.70.

CAT, computer adaptive test; DPDP, different patient-reported outcome different patient-reported outcome measure; DPSP, different patient-reported outcome same patient-reported outcome measure; PROMIS, patient-reported outcomes measurement information system; SF-36v2, Short Form-36 version 2; SPDP, same patient-reported outcome different patient-reported outcome measure.

a Construct validity was only determined for the PROMIS CATs physical function, pain interference, fatigue, anxiety, depression, and ability to participate in social roles and activities.

b Relevance, as in comparison with the general Dutch population, was only determined for the PROMIS CATs physical function, pain interference, anxiety, depression. ability to participate in social roles and activities, and satisfaction with social roles and activities.

## 4 Discussion

In this study, we demonstrate that the selected PROMIS measures are valid and reliable instruments to measure health-related quality of life in adults with VWD. The PROMIS CATs were considered feasible, since the number of completed items of the selected set of CATs was only slightly higher than that of the SF-36v2. In addition, the CATs had less floor and ceiling effects than the SF-36v2. This implies that the PROMIS CATs more adequately cover the range of functioning of adults with VWD. The PROMIS fixed scale global health was also considered feasible, with a lower number of completed items and similar floor and ceiling effects compared to the SF-36v2. The PROMIS CAT Anxiety and Depression had a good construct validity, while all other PROMIS CATs had an excellent construct validity. All PROMIS measures are reliable for use on the group level. Moreover, all studied PROMIS CATs and the short form anger can be used to draw reliable conclusions on an individual patient level.

Our study findings on the feasibility, construct validity, and reliability of the PROMIS CATs are consistent with previous studies performed in Dutch patients with hemophilia [21,22]. Regarding the relevance of the PROMIS CATs, our study findings are inconsistent with a previous study performed in Dutch patients with VWD. This previous study found that Dutch patients with VWD primarily experience reduced vitality compared with the general population [30]. Within this study, our population only reported slightly lower scores on the PROMIS CAT physical function compared to the Dutch general population [35,36,38]. Our study population consisted primarily of patients with a nonsevere bleeding phenotype. This may explain the difference with the previous study since patients with a severe bleeding disorder tend to have a more impacted health-related quality of life than patients with a nonsevere bleeding disorder [12,30]. In addition, our study population primarily consisted of females and elderly participants who both tend to score worse on physical functioning [15,57].

### 4.1 Strengths and limitations

This is the first study assessing the feasibility, measurement properties, and relevance of the PROMIS measures in patients with VWD. We followed the COSMIN guidelines, determined construct validity in a profound manner using the MTMM, and incorporated the latest methodology on reliability estimates [32,53,58].

A possible limitation of this study is the limited information on the feasibility of the PROMIS measures compared with the SF-36v2. In our study, feasibility was determined based on the number of items needed to complete the PROMIS measures compared with the SF-36v2 and the presence of floor or ceiling effects. Data on the time needed to complete the PROMIS CATs, short forms, fixed scale, and SF-36v2 were not available. Previous studies, however, reported that the median time needed to complete 7 PROMIS CATs was 10.2 minutes, while participants required a median of 5 minutes to complete the SF-36v2 [59,60]. These studies confirm our conclusion that the selected PROMIS CATs are slightly less feasible to complete than the SF-36v2. However, PROMIS CATs are more precise compared with the SF-36v2 due to the absence of ceiling effects and the limited occurrence of floor effects.

Other possible limitations of this study are related to sample size and generalizability of the results, partly due to the overall survey response rate of 33% and possible selection bias. The unequal distribution of patients across the 3 distinct types of inherited bleeding disorders, the limited information on patient characteristics (eg, race, ethnicity, or educational level), and the unequal distribution among the collected patient characteristics (ie, sex, disease severity, age group) limited our ability to determine the feasibility, measurement properties, and relevance of the PROMIS measures separately for each type of bleeding disorder and for disease severity. In addition, due to the limited number of included patients with IPFDs or RBDs and the heterogeneity within these 2 disease populations, we were only able to determine the feasibility, measurement properties, and relevance of the PROMIS measures for patients with VWD. Moreover, most patients had a nonsevere bleeding phenotype. The results of this study might therefore not be as representative for patients with a severe bleeding phenotype, patients receiving prophylactic treatment, or patients with IPFDs or RBDs. The rare nature of these bleeding disorders makes them inherently difficult to study. We therefore included the results of patients with IPFDs and RBDs in the Supplementary Methods, so that researchers could potentially use our results to inform future studies or pool results.

### 4.2 Clinical implications and future research

Our study shows that PROMIS measures are reliable and valid instruments to measure physical function, pain interference, fatigue, anxiety, depression, ability to participate in social roles and activities, satisfaction with social roles and activities, and anger in patients with VWD. In contrast to some domains of the SF-36v2 and the PROMIS fixed scale global health, all PROMIS CATs can be used to draw reliable conclusions on individual patient levels and can therefore be used in clinical practice. However, before PROMIS CATs can be implemented in research and routine care additional research should be performed on the feasibility of the PROMIS CATs. First, the number of PROMIS CAT items administrated is higher compared with the SF-36v2. Patients with no problems or disease symptoms have to answer a maximum of 12 questions to reach the CAT stopping rule. Adjustments to this stopping rule should be evaluated to explore if it is possible to reduce questionnaire burden [61]. Second, other aspects of feasibility such as the comprehensibility of the PROMIS CATs and SF-36v2 in patients with low health literacy and the cost for administration should be evaluated. Thirdly, additional research should be performed to evaluate the feasibility and measurement properties of the PROMIS measures in patients with IPFDs and RBDs. The results of patients with IPFDs and RBDs provide an indication that the PROMIS measures might also be feasible, reliable, and valid in these patient populations. However, additional research should be performed for confirmation. Lastly, not all PROMIS measures might be relevant for patients with VWD, IPFDs, and RBDs. Previous studies on important PROs for this patient population primarily identified bleeding symptoms that are not included in either the PROMIS measures or the SF-36v2 [62]. Further research should be performed to determine the best set of PROMIS CATs or short forms in combination with a bleeding disorder–specific questionnaire to enable measurement of all relevant PROs in this patient population. Preferably, (inter)national consensus should be achieved on this standard set of PROMs to facilitate the comparison between patient population, the general population, and other healthcare settings [23].

## 5 Conclusion

The studied PROMIS CATs, short form anger and fixed scale global health are a valid and reliable alternative for the SF-36v2 in patients with primarily nonsevere forms of VWD. Moreover, the PROMIS CATs and short form anger can be used to assess the health-related quality of life of this patient population on both group and individual patient levels and can therefore be used in clinical practice. The PROMIS fixed scale global health can be applied to compare the health-related quality of life of this patient population on group level. Further research is necessary to evaluate the feasibility and measurement properties of the PROMIS measures in adults with IPFDs and RBDs. Moreover, additional research should be performed to reduce the completion burden of the PROMIS CATs and to determine the optimal combination of generic PROMIS CATs with disease-specific questionnaires to measure PROs in patients with VWD, IPFDs, and RBDs.
