# Supplementary material for: Psychometrics of patient-reported outcomes measurement information system in von Willebrand disease, inherited platelet function disorders, and rare bleeding disorders
Source: Res Pract Thromb Haemost. 2024 Jun 13;8(4):102474. doi: 10.1016/j.rpth.2024.102474 (PMC11284954; doi:10.1016/j.rpth.2024.102474)
Supplement: Supplementary Table S1 [file mmc1.docx]

**Supplement to:**

**Psychometrics of patient-reported outcomes measurement information system in von Willebrand disease, inherited platelet function disorders, and rare bleeding disorders**

Evelien S. van Hoorn^1,*^, e.vanhoorn@erasmusmc.nl, Sterre P.E. Willems^2^, Wala Al Arashi^3^, Annick S. de Moor^4^, Calvin B. van Kwawegen^5^, Lorynn Teela^6,7,8^, Martijn A.H. Oude Voshaar^1^, Idske C.L. Kremer Hovinga^4^, Roger E.G. Schutgens^4^, Saskia E.M. Schols^2^, Frank W.G. Leebeek^5^, Lotte Haverman^6,7,8^, Marjon H. Cnossen^3^, Samantha C. Gouw^9^, and Hester F. Lingsma^1^, on behalf of the SYMPHONY consortium and the Dutch research group for PROMIS implementation in inherited bleeding disorders

^1^ Department of Public Health, Erasmus MC, University Medical Center Rotterdam, Rotterdam, The Netherlands

^2^ Department of Hematology, Radboud university medical center Nijmegen, Hemophilia Treatment Center Nijmegen-Eindhoven-Maastricht, Nijmegen, The Netherlands

^3^ Department of Pediatric Hematology, Erasmus MC Sophia Children's Hospital, University Medical Center Rotterdam, Rotterdam, The Netherlands

^4^ Center for Benign Hematology, Thrombosis and Hemostasis, Van Creveldkliniek University Medical Center Utrecht, Utrecht University, Utrecht, The Netherlands

^5^ Department of Hematology, Erasmus MC, University Medical Center Rotterdam, Rotterdam, The Netherlands

^6^ Child and Adolescent Psychiatry & Psychosocial Care, Emma Children’s Hospital, Amsterdam UMC location University of Amsterdam, Amsterdam, The Netherlands

^7^ Public Health, Mental health and Digital health, Amsterdam UMC location University of Amsterdam, Amsterdam, The Netherlands.

^8^ Reproduction and Development, Child development, Amsterdam UMC location University of Amsterdam, Amsterdam, The Netherlands

^9^ Pediatric Hematology, Emma Children’s Hospital, Amsterdam UMC location University of Amsterdam, Amsterdam, The Netherlands

**Correspondence** Evelien .S. van Hoorn, Department of Public Health, Erasmus MC, University Medical Centre Rotterdam, Dr. Molewaterplein 40, 3015 GD Rotterdam, The Netherlands.

**Content**

**Inclusion criteria...............................................................................................................3**

**Results in adults with von Willebrand disease..................................................................4**

Table S1: Additional patient characteristics.........................................................................4

Table S2: Correlation matrix between PROMIS CATs and the SF-36v2 domains..................5

Table S3: Overview of the skewness, inter-item correlation and reliability of the SF-36v...6

Table S4: Overview of the T-scores and standard errors......................................................6

**Results in adults with inherited platelet function disorders..............................................7**

Table S5: Number of completed items, floor and ceiling effects, and reliability of the

PROMIS measures.................................................................................................7

Table S6: Number of completed items, floor and ceiling effects, and reliability of the

SF-36v2..................................................................................................................7

Table S7: Summary of the correlation matrix for construct validity assessment.................8

Table S8: Distribution of PROMIS CATs scores according to the Dutch thresholds............. 9

Table S9: Correlation matrix between PROMIS CATs and the SF-36v2 domains................10

Table S10: Overview of the skewness, inter-item correlation and reliability of the

SF-36v2...............................................................................................................11

Table S11: Overview of the T-scores and standard errors..................................................11

**Results in adults with rare bleeding disorders.................................................................12**

Table S12: Additional patient characteristics............................... ......................................12

Table S13: Number of completed items, floor and ceiling effects, and reliability of the

PROMIS measures.............................................................................................12

Table S14: Number of completed items, floor and ceiling effects, and reliability of the

SF-36v2..............................................................................................................13

Table S15: Summary of the correlation matrix for construct validity assessment..............13

Table S16: Distribution of PROMIS CATs scores according to the Dutch thresholds...........14

Table S17: Correlation matrix between PROMIS CATs and the SF-36v2 domains...............15

Table S18: Overview of the skewness, inter-item correlation and reliability of the

SF-36v2...............................................................................................................16

Table S19: Overview of the T-scores and standard errors...................................................16

**Inclusion criteria**

Patients with von Willebrand disease were invited to participate if they met the following criteria:

- Historically lowest VWF:Ag and/or VWF:RCo and/or VWF:CB ≤ 0.30 IU/mL and/or FVIII:C ≤ 0.40 IU/mL (type 2N)
- Participated for two years in the Von Willebrand in the Netherlands – Prospective (WiN-Pro) study
- Age ≥ 18 years old
- Receives treatment at Erasmus MC, Erasmus University Medical Center, Rotterdam, the Netherlands

Patients with an inherited platelet function disorder were invited to participate if they met the following criteria:

- Participation in the Thrombocytopathy in the Netherlands (TiN) study and presence of permission to be contacted for follow-up studies
- Patients with a confirmed congenital blood platelet disorders as defined by the TiN study; patients where an abnormal platelet function was found on at least two occasions, of which one was in diagnostics laboratory during the TiN study.
- Age ≥ 18 years old

Rare bleeding disorders (RBDs) were defined in the Rare Bleeding disorder in the Netherlands study (2017-2019) as deficiencies of factor (F) II, FV, combined FV&FVIII, FVII, FX, FXI, FXIII, FV Amsterdam, and disorders of fibrinolysis: plasminogen activator type 1 (PAI-1) deficiency, alpha-2- antiplasmin (A2AP) deficiency, and hyperfibrinolysis. Patients with an RBD were invited to participate if they met the following criteria:

- Participation in the Rare Bleeding disorder in the Netherlands (RBiN) study and presence of permission to be contacted for follow-up studies;
- Permission to use personal email address for study participation invitation via https://promis-symphony.nl/;
- Age ≥ 18 years old;
- Receives treatment at Hemophilia Treatment Center Nijmegen-Eindhoven-Maastricht, location Radboud university medical center, Radboudumc, Nijmegen, the Netherlands.

**Results in adults with von Willebrand disease**

Table S1: Additional patient characteristics

|  | | **von Willebrand disease**  **N= 67** |
| --- | --- | --- |
| ***Type of VWD*** | |  |
|  | Type 1 | 43 (64%) |
|  | Type 2 | 22 (33%) |
|  | Type 3 | 2 (3%) |

Table S2: Correlation matrix between PROMIS CATs and the SF-36v2 domains in adults with von Willebrand disease.

|  | **SF-36v2** | | | | | | | | **PROMIS Computer Adaptive Tests** | | | | | |
| --- | --- | --- | --- | --- | --- | --- | --- | --- | --- | --- | --- | --- | --- | --- |
| **SF-36v2** | **PF** | **RP** | **RE** | **VT** | **MH** | **SF** | **BP** | **GH** | **PF** | **PI** | **FT** | **AN** | **DE** | **AP** |
| Physical functioning | **1** |  |  |  |  |  |  |  |  |  |  |  |  |  |
| Role limitations due to physical health problems | 0.46 | **1** |  |  |  |  |  |  |  |  |  |  |  |  |
| Role limitations due to emotional problems | 0.39 | 0.68 | **1** |  |  |  |  |  |  |  |  |  |  |  |
| Vitality | 0.64 | 0.49 | 0.33 | **1** |  |  |  |  |  |  |  |  |  |  |
| Mental health | 0.44 | 0.42 | 0.32 | 0.63 | **1** |  |  |  |  |  |  |  |  |  |
| Social functioning | 0.67 | 0.44 | 0.43 | 0.59 | 0.60 | **1** |  |  |  |  |  |  |  |  |
| Bodily pain | 0.76 | 0.43 | 0.28 | 0.58 | 0.58 | 0.61 | **1** |  |  |  |  |  |  |  |
| General health | 0.68 | 0.37 | 0.26 | 0.73 | 0.59 | 0.60 | 0.61 | **1** |  |  |  |  |  |  |
| **PROMIS Computer Adaptive Tests** | | |  |  |  |  |  |  |  |  |  |  |  |  |
| Physical function | 0.92 | 0.43 | 0.35 | 0.66 | 0.41 | 0.69 | 0.74 | 0.71 | **1** |  |  |  |  |  |
| Pain interference | -0.69 | -0.33 | -0.22 | -0.50 | -0.48 | -0.57 | -0.92 | -0.52 | -0.70 | **1** |  |  |  |  |
| Fatigue | -0.64 | -0.36 | -0.23 | -0.86 | -0.56 | -0.56 | -0.59 | -0.69 | -0.64 | 0.51 | **1** |  |  |  |
| Anxiety | -0.42 | -0.26 | -0.09 | -0.58 | -0.67 | -0.40 | -0.53 | -0.52 | -0.41 | 0.50 | 0.66 | **1** |  |  |
| Depression | -0.42 | -0.34 | -0.17 | -0.58 | -0.71 | -0.45 | -0.55 | -0.44 | -0.39 | 0.50 | 0.53 | 0.74 | **1** |  |
| Ability to participate in social roles and activities | 0.67 | 0.41 | 0.30 | 0.65 | 0.49 | 0.67 | 0.59 | 0.63 | 0.69 | -0.59 | -0.61 | -0.53 | -0.51 | **1** |

Abbreviations: PF, physical functioning; RP, role limitations due to physical health problems; RE, role limitations due to emotional problems; VT, vitality; MH, mental health; SF, social functioning; BP, bodily pain; GH, general health; PI, pain interference; FT, fatigue; AN, anxiety; DE, depression; AP, ability to participate in social roles and activities; SF, short-form; PROMIS, patient-reported outcomes measurement information system.

Table S3: Overview of the skewness, inter-item correlation and reliability of the SF-36v2 in adults with von Willebrand disease.

|  |  |  |  | **Score** | |  | **Correlation** | | **Reliability** | |
| --- | --- | --- | --- | --- | --- | --- | --- | --- | --- | --- |
| **Instruments** | | **N** | **N. of items** | **Mean** | **SD** | **Skew** | **Min** | **Max** | **α** | **GLB** |
| ***SF-36v2*** | |  |  |  |  |  |  |  |  |  |
|  | Physical functioning | 63 | 10 | 71.0 | 28.9 | -0.97 | 0.31 | 0.82 | 0.94 | 0.98 |
|  | Role limitations due to physical health problems | 63 | 4 | 92.1 | 23.2 | -3.13 | 0.46 | 0.78 | 0.88 | 0.93 |
|  | Role limitations due to emotional problems | 63 | 3 | 94.7 | 21.8 | -3.95 | 0.86 | - | 0.96 | 0.96 |
|  | Vitality | 63 | 4 | 62.1 | 16.9 | -0.04 | 0.40 | 0.65 | 0.82 | 0.89 |
|  | Mental health | 63 | 5 | 71.0 | 13.2 | -0.80 | 0.30 | 0.73 | 0.85 | 0.92 |
|  | Social functioning | 63 | 2 | 78.6 | 24.6 | -1.15 | 0.67 | - | 0.80 | 0.80 |
|  | (Bodily) pain | 63 | 2 | 70.9 | 27.0 | -0.55 | 0.83 | - | 0.91 | 0.91 |
|  | General health | 63 | 5 | 58.5 | 25.0 | -0.76 | 0.39 | 0.76 | 0.87 | 0.91 |
|  | Physical component score | 63 | 21 | 46.0 | 10.6 | -0.56 | 0.36 | 0.68 | 0.83 | 0.86 |
|  | Mental component score | 63 | 14 | 51.5 | 6.2 | -1.03 | 0.29 | 0.66 | 0.77 | 0.85 |

Abbreviations: α, Cronbach’s alpha; GLB, greatest lower bound; max, maximum; min, minimum; SD, standard deviation.

Table S4: Overview of the T-scores and standard errors in adults with von Willebrand disease.

|  |  |  | **T-score** | | | | **Standard errors** | | |  |
| --- | --- | --- | --- | --- | --- | --- | --- | --- | --- | --- |
| **PROMIS item bank** | | **N** | **Mean** | **SD** | **Min** | **Max** | **Mean** | **SD** | **Min** | **Max** |
| ***Computer Adaptive Tests*** | |  |  |  |  |  |  |  |  |  |
|  | Physical function | 67 | 47.2 | 10.0 | 19.8 | 64.7 | 2.1 | 0.3 | 1.5 | 3.0 |
|  | Pain interference | 67 | 51.3 | 9.6 | 41.0 | 75.7 | 2.7 | 1.2 | 1.0 | 4.1 |
|  | Fatigue | 66 | 50.4 | 10.8 | 28.8 | 78.1 | 2.1 | 0.3 | 1.7 | 3.2 |
|  | Anxiety | 66 | 50.2 | 7.5 | 36.4 | 70.5 | 2.4 | 0.6 | 2.0 | 4.0 |
|  | Depression | 65 | 49.5 | 6.9 | 39.0 | 66.3 | 2.5 | 0.7 | 1.9 | 4.1 |
|  | Ability to participate in social roles and activities | 64 | 53.2 | 7.8 | 37.9 | 64.9 | 2.2 | 0.5 | 1.6 | 3.4 |
|  | Satisfaction with social roles and activities | 64 | 50.9 | 7.0 | 38.1 | 65.9 | 2.0 | 0.5 | 1.5 | 3.4 |
| ***Short form*** | |  |  |  |  |  |  |  |  |  |
|  | Anger | 63 | 49.1 | 8.3 | 36.3 | 68.1 | 3.2 | 0.4 | 2.8 | 4.0 |
| ***Fixed scale*** | |  |  |  |  |  |  |  |  |  |
|  | Global Health – physical health | 62 | 39.9 | 6.6 | 29.6 | 57.7 | 4.2 | 0.2 | 4.1 | 4.9 |
|  | Global Health – mental health | 62 | 37.2 | 9.1 | 21.2 | 53.3 | 3.8 | 0.3 | 3.6 | 4.6 |

Abbreviations: max, maximum; min, minimum; SD, standard deviation

**Results in adults with inherited platelet function disorders**

Table S5: Number of completed items, floor and ceiling effects, and reliability of the PROMIS measures in adults with inherited platelet function disorders.

|  |  |  | **No. of items** | | | | **Floor** | **Ceiling** | **Reliability** |
| --- | --- | --- | --- | --- | --- | --- | --- | --- | --- |
| **PROMIS measure** | | **N** | **Mean** | **SD** | **Min** | **Max** | **%** | **%** | **Coefficient** |
| ***Computer Adaptive Tests*** | |  |  |  |  |  |  |  |  |
|  | Physical function | 18 | 5.8 | 2.7 | 4 | 12 | 0% | 0% | 0.96 |
|  | Pain interference | 18 | 6.0 | 3.4 | 4 | 12 | 22% | 0% | 0.94 |
|  | Fatigue | 18 | 5.7 | 1.9 | 4 | 12 | 0% | 0% | 0.96 |
|  | Anxiety | 18 | 8.9 | 2.3 | 5 | 12 | 0% | 0% | 0.95 |
|  | Depression | 18 | 6.6 | 1.6 | 4 | 9 | 11% | 0% | 0.94 |
|  | Ability to participate in social roles and activities | 18 | 5.2 | 2.4 | 4 | 12 | 0% | 6% | 0.96 |
|  | Satisfaction with social roles and activities | 18 | 4.6 | 1.9 | 4 | 12 | 0% | 6% | 0.96 |
| ***PROMIS Short Form*** | |  |  |  |  |  |  |  |  |
|  | Anger | 18 | 5 | - | 5 | 5 | 11% | 0% | 0.90 |
| ***PROMIS Fixed scale*** | |  |  |  |  |  |  |  |  |
|  | Global Health – physical health | 17 | 4 | - | 4 | 4 | 0% | 0% | 0.82 |
|  | Global Health – mental health | 17 | 4 | - | 4 | 4 | 0% | 0% | 0.87 |

Table S6: Number of completed items, floor and ceiling effects, and reliability of the SF-36v2 in adults with inherited platelet function disorders.

|  |  |  |  | **Floor** | **Ceiling** | **Reliability** |  |
| --- | --- | --- | --- | --- | --- | --- | --- |
| **Instruments** | | **N** | **No. of items** | **%** | **%** | **Cronbach’s α** | **Greatest lower bound** |
| ***SF-36v2*** | |  |  |  |  |  |  |
|  | Physical functioning | 18 | 10 | 6% | 11% | 0.94 | 0.99 |
|  | Role limitations due to physical health problems | 18 | 4 | 6% | 72% | 0.79 | 0.84 |
|  | Role limitations due to emotional problems | 18 | 3 | 0% | 89% | N/A | N/A |
|  | Vitality | 18 | 4 | 0% | 0% | 0.91 | 0.96 |
|  | Mental health | 18 | 5 | 0% | 0% | 0.75 | 0.90 |
|  | Social functioning | 18 | 2 | 0% | 11% | 0.85 | 0.85 |
|  | Bodily pain | 18 | 2 | 0% | 22% | 0.91 | 0.91 |
|  | General health | 18 | 5 | 0% | 0% | 0.79 | 0.88 |
|  | Physical component score | 18 | 21 | 0% | 0% | 0.79 | 0.89 |
|  | Mental component score | 18 | 14 | 0% | 0% | 0.76 | 0.89 |

Table S7: Summary of the correlation matrix for construct validity assessment in adults with inherited platelet function disorders.

|  | **Same PRO, same PROM** | **Same PRO, different PROM^*^** | **Different PRO, same PROM** | | **Different PRO, different PROM** | |
| --- | --- | --- | --- | --- | --- | --- |
| **PROMIS Computer Adaptive Tests** | **Reliability coefficient** | **Corr. ^†^** | **Min corr. ^†^** | **Max**  **corr.^†^** | **Min**  **corr. ^†^** | **Max**  **corr. ^†^** |
| Physical function | 0.96 | 0.85 | 0.44 | 0.84 | 0.17 | 0.82 |
| Pain interference | 0.94 | 0.93 | 0.22 | 0.82 | 0.12 | 0.78 |
| Fatigue | 0.96 | 0.86 | 0.27 | 0.67 | 0.19 | 0.68 |
| Anxiety | 0.95 | 0.35 | 0.22 | 0.53 | 0.01 | 0.39 |
| Depression | 0.94 | 0.60 | 0.27 | 0.57 | 0.23 | 0.66 |
| Ability to participate in social roles and activities | 0.96 | 0.85 | 0.44 | 0.84 | 0.22 | 0.75 |

**^*^** CAT physical function was compared with SF-36v2 physical functioning, CAT pain interference was compared with SF-36v2 bodily pain, CAT fatigue was compared with SF-36v2 vitality, CAT anxiety and depression were compared to SF-36v2 mental health, CAT ability to participate in social roles and activities was compared with SF-36v2 social functioning.

^†^ Absolute correlations

Abbreviations: CATs, Computer Adaptive Tests; corr., correlation; Max, maximum; PROMIS, Patient Reported Outcomes Measurement Information System; SF, short form.

Table S8: Distribution of PROMIS CATs scores according to the Dutch thresholds in adults with inherited platelet function disorders and the Dutch general population.

|  |  | **Within normal limits** | **Mild symptoms** | **Moderate symptoms** | **Severe symptoms** |
| --- | --- | --- | --- | --- | --- |
| **Physical function** | |  |  |  |  |
|  | Study population | 61% | 17% | 17% | 5% |
|  | Dutch general population | 68% | 13% | 17% | 2% |
| **Pain Interference** | |  |  |  |  |
|  | Study population | 66% | 6% | 22% | 6% |
|  | Dutch general population | 66% | 19% | 14% | 1% |
| **Anxiety** | |  |  |  |  |
|  | Study population | 67% | 28% | 0% | 5% |
|  | Dutch general population | 70% | 14% | 15% | 1% |
| **Depression** | |  |  |  |  |
|  | Study population | 61% | 39% | 0% | 0% |
|  | Dutch general population | 71% | 15% | 13% | 1% |
| **Ability to participate in social roles and activities** | | |  |  |  |
|  | Study population | 56% | 22% | 22% | 0% |
|  | Dutch general population | 68% | 19% | 11% | 2% |
| **Satisfaction with social roles and activities** | | |  |  |  |
|  | Study population | 78% | 17% | 5% | 0% |
|  | Dutch general population | 73% | 11% | 12% | 4% |

Table S9: Correlation matrix between PROMIS CATs and the SF-36v2 domains in adults with inherited platelet function disorders

|  | **SF-36v2** | | | | | | | | **PROMIS Computer Adaptive Tests** | | | | | |
| --- | --- | --- | --- | --- | --- | --- | --- | --- | --- | --- | --- | --- | --- | --- |
| **SF-36v2** | **PF** | **RP** | **RE** | **VT** | **MH** | **SF** | **BP** | **GH** | **PF** | **PI** | **FT** | **AN** | **DE** | **AP** |
| Physical functioning | **1** |  |  |  |  |  |  |  |  |  |  |  |  |  |
| Role limitations due to physical health problems | 0.41 | **1** |  |  |  |  |  |  |  |  |  |  |  |  |
| Role limitations due to emotional problems | 0.30 | 0.56 | **1** |  |  |  |  |  |  |  |  |  |  |  |
| Vitality | 0.52 | 0.69 | 0.26 | **1** |  |  |  |  |  |  |  |  |  |  |
| Mental health | 0.37 | 0.07 | 0.28 | 0.35 | **1** |  |  |  |  |  |  |  |  |  |
| Social functioning | 0.59 | 0.58 | 0.40 | 0.83 | 0.39 | **1** |  |  |  |  |  |  |  |  |
| Bodily pain | 0.75 | 0.51 | 0.43 | 0.47 | 0.20 | 0.49 | **1** |  |  |  |  |  |  |  |
| General health | 0.57 | 0.49 | 0.30 | 0.64 | 0.25 | 0.73 | 0.26 | **1** |  |  |  |  |  |  |
| **PROMIS Computer Adaptive Tests** | | |  |  |  |  |  |  |  |  |  |  |  |  |
| Physical function | 0.85 | 0.65 | 0.34 | 0.62 | 0.17 | 0.69 | 0.82 | 0.62 | **1** |  |  |  |  |  |
| Pain interference | -0.78 | -0.56 | -0.41 | -0.38 | -0.12 | -0.44 | -0.93 | -0.31 | -0.82 | **1** |  |  |  |  |
| Fatigue | -0.53 | -0.58 | -0.19 | -0.86 | -0.08 | -0.68 | -0.52 | -0.51 | -0.61 | 0.43 | **1** |  |  |  |
| Anxiety | -0.37 | 0.01 | -0.15 | -0.27 | -0.35 | -0.31 | -0.39 | -0.39 | -0.44 | 0.22 | 0.34 | **1** |  |  |
| Depression | -0.66 | -0.23 | -0.48 | -0.43 | -0.60 | -0.55 | -0.52 | -0.56 | -0.55 | 0.50 | 0.27 | 0.53 | **1** |  |
| Ability to participate in social roles and activities | 0.65 | 0.64 | 0.37 | 0.75 | 0.22 | 0.85 | 0.65 | 0.72 | 0.84 | -0.57 | -0.67 | -0.44 | -0.57 | **1** |

Abbreviations: PF, physical functioning; RP, role limitations due to physical health problems; RE, role limitations due to emotional problems; VT, vitality; MH, mental health; SF, social functioning; BP, bodily pain; GH, general health; PI, pain interference; FT, fatigue; AN, anxiety; DE, depression; AP, ability to participate in social roles and activities; SF, short-form; PROMIS, patient-reported outcomes measurement information system.

Table S10: Overview of the skewness, inter-item correlation and reliability of the SF-36v2 in adults with inherited platelet function disorders.

|  |  |  |  | **Score** | |  | **Correlation** | | **Reliability** | |
| --- | --- | --- | --- | --- | --- | --- | --- | --- | --- | --- |
| **Instruments** | | **N** | **N. of items** | **Mean** | **SD** | **Skew** | **Min** | **Max** | **α** | **GLB** |
| ***SF-36v2*** | |  |  |  |  |  |  |  |  |  |
|  | Physical functioning | 18 | 10 | 68.3 | 29.3 | -0.67 | 0.34 | 0.94 | 0.94 | 0.99 |
|  | Role limitations due to physical health problems | 18 | 4 | 87.5 | 26.1 | -2.20 | 0.45 | 0.54 | 0.79 | 0.84 |
|  | Role limitations due to emotional problems | 18 | 3 | 96.3 | 10.8 | -2.27 | N/A | N/A | N/A | N/A |
|  | Vitality | 18 | 4 | 50.8 | 21.0 | -0.12 | 0.61 | 0.89 | 0.91 | 0.96 |
|  | Mental health | 18 | 5 | 63.6 | 13.6 | -0.06 | 0.07 | 0.66 | 0.75 | 0.90 |
|  | Social functioning | 18 | 2 | 65.3 | 21.7 | 0 | 0.74 | - | 0.85 | 0.85 |
|  | (Bodily) pain | 18 | 2 | 67.2 | 27.3 | -0.44 | 0.84 | - | 0.91 | 0.91 |
|  | General health | 18 | 5 | 48.3 | 21.1 | 0.48 | 0.11 | 0.81 | 0.79 | 0.88 |
|  | Physical component score | 18 | 21 | 44.0 | 11.2 | -0.42 | 0.21 | 0.71 | 0.79 | 0.89 |
|  | Mental component score | 18 | 14 | 47.5 | 6.3 | -0.39 | 0.23 | 0.85 | 0.76 | 0.89 |

Abbreviations: α, Cronbach’s alpha; GLB, greatest lower bound; max, maximum; min, minimum; SD, standard deviation.

Table S11: Overview of the T-scores and standard errors in adults with inherited platelet function disorders.

|  |  |  | **T-score** | | | | **Standard errors** | | |  |
| --- | --- | --- | --- | --- | --- | --- | --- | --- | --- | --- |
| **PROMIS item bank** | | **N** | **Mean** | **SD** | **Min** | **Max** | **Mean** | **SD** | **Min** | **Max** |
| ***Computer Adaptive Tests*** | |  |  |  |  |  |  |  |  |  |
|  | Physical function | 18 | 47.4 | 9.2 | 26.4 | 61.0 | 2.0 | 0.2 | 1.4 | 2.4 |
|  | Pain interference | 18 | 55.8 | 10.0 | 41.0 | 74.7 | 2.1 | 1.2 | 1.0 | 4.1 |
|  | Fatigue | 18 | 55.3 | 10.7 | 30.1 | 74.9 | 2.0 | 0.2 | 1.8 | 2.8 |
|  | Anxiety | 18 | 51.7 | 7.4 | 40.6 | 70.2 | 2.2 | 0.3 | 2.0 | 3.1 |
|  | Depression | 18 | 51.9 | 5.7 | 39.0 | 59.3 | 2.4 | 0.6 | 1.9 | 4.1 |
|  | Ability to participate in social roles and activities | 18 | 47.8 | 8.1 | 33.5 | 64.9 | 2.0 | 0.4 | 1.7 | 3.4 |
|  | Satisfaction with social roles and activities | 18 | 47.8 | 7.1 | 34.5 | 65.9 | 2.0 | 0.4 | 1.5 | 3.4 |
| ***Short form*** | |  |  |  |  |  |  |  |  |  |
|  | Anger | 18 | 52.4 | 7.7 | 36.3 | 63.2 | 3.1 | 0.4 | 2.8 | 4.0 |
| ***Fixed scale*** | |  |  |  |  |  |  |  |  |  |
|  | Global Health – physical health | 17 | 43.6 | 4.7 | 37.4 | 54.1 | 4.3 | 0.2 | 4.1 | 4.7 |
|  | Global Health – mental health | 17 | 42.4 | 6.5 | 31.3 | 53.3 | 3.6 | 0.1 | 3.6 | 3.7 |

Abbreviations: max, maximum; min, minimum; SD, standard deviation

**Results in adults with rare bleeding disorders**

Table S12: Additional patient characteristics

|  | | **Rare bleeding disorders**  **N=27** |
| --- | --- | --- |
| ***Type of rare bleeding disorder*** | |  |
|  | Afibrinogenemia | 1 (3.8%) |
|  | Afibrinogenemia (family member of) | 2 (7.7%) |
|  | Hypofibrinogenemia | 2 (7.7%) |
|  | Dysfibrinogenemia | 1 (3.8%) |
|  | FII deficiency | 2 (7.7%) |
|  | FV deficiency | 1 (3.8%) |
|  | FVII deficiency | 3 (12%) |
|  | FXI deficiency | 3 (12%) |
|  | Alpha2-antiplasmin deficiency | 1 (3.8%) |
|  | Plasminogen activator inhibitor type 1 deficiency | 5 (19%) |
|  | Hyperfibrinolysis | 5 (19%) |
| ***Educational level*** | |  |
|  | Lower education | 3 (14%) |
|  | Secondary education | 8 (36%) |
|  | Higher education | 11 (50%) |
|  | Missing | 4 |

Table S13: Number of completed items, floor and ceiling effects, and reliability of the PROMIS measures in adults with rare bleeding disorders.

|  |  |  | **No. of items** | | | | **Floor** | **Ceiling** | **Reliability** |
| --- | --- | --- | --- | --- | --- | --- | --- | --- | --- |
| **PROMIS measure** | | **N** | **Mean** | **SD** | **Min** | **Max** | **%** | **%** | **Coefficient** |
| ***Computer Adaptive Tests*** | |  |  |  |  |  |  |  |  |
|  | Physical function | 26 | 6.6 | 3.0 | 4 | 12 | 0% | 4% | 0.95 |
|  | Pain interference | 26 | 7.8 | 3.9 | 4 | 12 | 38% | 0% | 0.92 |
|  | Fatigue | 26 | 6.2 | 2.7 | 4 | 12 | 4% | 0% | 0.95 |
|  | Anxiety | 26 | 9.1 | 2.4 | 6 | 12 | 15% | 0% | 0.93 |
|  | Depression | 26 | 7.9 | 1.5 | 5 | 9 | 31% | 0% | 0.91 |
|  | Ability to participate in social roles and activities | 26 | 6.7 | 2.8 | 4 | 12 | 0% | 12% | 0.95 |
|  | Satisfaction with social roles and activities | 26 | 6.7 | 3.5 | 4 | 12 | 0% | 8% | 0.95 |
| ***PROMIS Short Form*** | |  |  |  |  |  |  |  |  |
|  | Anger | 26 | 5 | - | 5 | 5 | 19% | 0% | 0.89 |
| ***PROMIS Fixed scale*** | |  |  |  |  |  |  |  |  |
|  | Global Health – physical health | 26 | 4 | - | 4 | 4 | 0% | 0% | 0.82 |
|  | Global Health – mental health | 26 | 4 | - | 4 | 4 | 23% | 0% | 0.85 |

Table S14: Number of completed items, floor and ceiling effects, and reliability of the SF-36v2 in adults with rare bleeding disorders.

|  |  |  |  | **Floor** | **Ceiling** | **Reliability** |  |
| --- | --- | --- | --- | --- | --- | --- | --- |
| **Instruments** | | **N** | **No. of items** | **%** | **%** | **Cronbach’s α** | **Greatest lower bound** |
| ***SF-36v2*** | |  |  |  |  |  |  |
|  | Physical functioning | 26 | 10 | 0% | 19% | 0.94 | 0.98 |
|  | Role limitations due to physical health problems | 26 | 4 | 0% | 96% | N/A | N/A |
|  | Role limitations due to emotional problems | 26 | 3 | 0% | 92% | N/A | N/A |
|  | Vitality | 26 | 4 | 0% | 0% | 0.87 | 0.92 |
|  | Mental health | 26 | 5 | 0% | 0% | 0.83 | 0.93 |
|  | Social functioning | 26 | 2 | 0% | 54% | 0.84 | 0.89 |
|  | Bodily pain | 26 | 2 | 0% | 19% | 0.96 | 0.96 |
|  | General health | 26 | 5 | 0% | 4% | 0.87 | 0.93 |
|  | Physical component score | 26 | 21 | 0% | 0% | 0.75 | 0.86 |
|  | Mental component score | 26 | 14 | 0% | 0% | 0.72 | 0.88 |

Table S15: Summary of the correlation matrix for construct validity assessment in adults with rare bleeding disorders.

|  | **Same PRO, same PROM** | **Same PRO, different PROM^*^** | **Different PRO, same PROM** | | **Different PRO, different PROM** | |
| --- | --- | --- | --- | --- | --- | --- |
| **PROMIS Computer Adaptive Tests** | **Reliability coefficient** | **Corr. ^†^** | **Min corr. ^†^** | **Max**  **corr.^†^** | **Min**  **corr. ^†^** | **Max**  **corr. ^†^** |
| Physical function | 0.95 | 0.92 | 0.45 | 0.80 | 0.19 | 0.81 |
| Pain interference | 0.92 | 0.83 | 0.32 | 0.63 | 0.03 | 0.66 |
| Fatigue | 0.95 | 0.83 | 0.37 | 0.63 | 0.21 | 0.76 |
| Anxiety | 0.93 | 0.73 | 0.32 | 0.81 | 0.04 | 0.60 |
| Depression | 0.91 | 0.63 | 0.37 | 0.81 | 0.10 | 0.49 |
| Ability to participate in social roles and activities | 0.95 | 0.85 | 0.37 | 0.80 | 0.19 | 0.80 |

**^*^** CAT physical function was compared with SF-36v2 physical functioning, CAT pain interference was compared with SF-36v2 bodily pain, CAT fatigue was compared with SF-36v2 vitality, CAT anxiety and depression were compared to SF-36v2 mental health, CAT ability to participate in social roles and activities was compared with SF-36v2 social functioning.

^†^ Absolute correlations

Abbreviations: CATs, Computer Adaptive Tests; corr., correlation; Max, maximum; PROMIS, Patient Reported Outcomes Measurement Information System; SF, short form.

Table S16: Distribution of PROMIS CATs scores according to the Dutch thresholds in adults with rare bleeding disorders and the Dutch general population.

|  |  | **Within normal limits** | **Mild symptoms** | **Moderate symptoms** | **Severe symptoms** |
| --- | --- | --- | --- | --- | --- |
| **Physical function** | |  |  |  |  |
|  | Study population | 61% | 27% | 12% | 0% |
|  | Dutch general population | 68% | 13% | 17% | 2% |
| **Pain Interference** | |  |  |  |  |
|  | Study population | 69% | 19% | 8% | 4% |
|  | Dutch general population | 66% | 19% | 14% | 1% |
| **Anxiety** | |  |  |  |  |
|  | Study population | 77% | 19% | 4% | 0% |
|  | Dutch general population | 70% | 14% | 15% | 1% |
| **Depression** | |  |  |  |  |
|  | Study population | 85% | 11% | 4% | 0% |
|  | Dutch general population | 71% | 15% | 13% | 1% |
| **Ability to participate in social roles and activities** | | |  |  |  |
|  | Study population | 76% | 12% | 12% | 0% |
|  | Dutch general population | 68% | 19% | 11% | 2% |
| **Satisfaction with social roles and activities** | | |  |  |  |
|  | Study population | 88% | 8% | 4% | 0% |
|  | Dutch general population | 73% | 11% | 12% | 4% |

Table S17: Correlation matrix between PROMIS CATs and the SF-36v2 domains in adults with rare bleeding disorders.

|  | **SF-36v2** | | | | | | | | **PROMIS Computer Adaptive Tests** | | | | | |
| --- | --- | --- | --- | --- | --- | --- | --- | --- | --- | --- | --- | --- | --- | --- |
| **SF-36v2** | **PF** | **RP** | **RE** | **VT** | **MH** | **SF** | **BP** | **GH** | **PF** | **PI** | **FT** | **AN** | **DE** | **AP** |
| Physical functioning | **1** |  |  |  |  |  |  |  |  |  |  |  |  |  |
| Role limitations due to physical health problems | 0.12 | **1** |  |  |  |  |  |  |  |  |  |  |  |  |
| Role limitations due to emotional problems | -0.01 | 0.69 | **1** |  |  |  |  |  |  |  |  |  |  |  |
| Vitality | 0.70 | 0.34 | 0.16 | **1** |  |  |  |  |  |  |  |  |  |  |
| Mental health | 0.30 | 0.04 | 0.17 | 0.57 | **1** |  |  |  |  |  |  |  |  |  |
| Social functioning | 0.77 | 0.25 | 0.24 | 0.86 | 0.46 | **1** |  |  |  |  |  |  |  |  |
| Bodily pain | 0.70 | 0.18 | -0.08 | 0.67 | 0.13 | 0.61 | **1** |  |  |  |  |  |  |  |
| General health | 0.51 | 0.31 | 0.15 | 0.72 | 0.14 | 0.63 | 0.66 | **1** |  |  |  |  |  |  |
| **PROMIS Computer Adaptive Tests** | | |  |  |  |  |  |  |  |  |  |  |  |  |
| Physical function | 0.92 | 0.20 | 0.19 | 0.71 | 0.41 | 0.81 | 0.60 | 0.52 | **1** |  |  |  |  |  |
| Pain interference | -0.66 | -0.29 | -0.05 | -0.56 | -0.03 | -0.47 | -0.83 | -0.52 | -0.53 | **1** |  |  |  |  |
| Fatigue | -0.63 | -0.28 | -0.21 | -0.83 | -0.26 | -0.73 | -0.64 | -0.76 | -0.60 | 0.63 | **1** |  |  |  |
| Anxiety | -0.50 | 0.04 | -0.06 | -0.60 | -0.73 | -0.49 | -0.25 | -0.22 | -0.45 | 0.32 | 0.47 | **1** |  |  |
| Depression | -0.39 | -0.14 | -0.26 | -0.49 | -0.63 | -0.43 | -0.18 | -0.10 | -0.45 | 0.37 | 0.37 | 0.81 | **1** |  |
| Ability to participate in social roles and activities | 0.75 | 0.28 | 0.19 | 0.80 | 0.53 | 0.85 | 0.48 | 0.56 | 0.80 | -0.37 | -0.63 | -0.59 | -0.56 | **1** |

Abbreviations: PF, physical functioning; RP, role limitations due to physical health problems; RE, role limitations due to emotional problems; VT, vitality; MH, mental health; SF, social functioning; BP, bodily pain; GH, general health; PI, pain interference; FT, fatigue; AN, anxiety; DE, depression; AP, ability to participate in social roles and activities; SF, short-form; PROMIS, patient-reported outcomes measurement information system.

Table S18: Overview of the skewness, inter-item correlation and reliability of the SF-36v2 in adults with rare bleeding disorders.

|  |  |  |  | **Score** | |  | **Correlation** | | **Reliability** | |
| --- | --- | --- | --- | --- | --- | --- | --- | --- | --- | --- |
| **Instruments** | | **N** | **N. of items** | **Mean** | **SD** | **Skew** | **Min** | **Max** | **α** | **GLB** |
| ***SF-36v2*** | |  |  |  |  |  |  |  |  |  |
|  | Physical functioning | 26 | 10 | 79.2 | 24.6 | -1.27 | 0.45 | 0.94 | 0.94 | 0.98 |
|  | Role limitations due to physical health problems | 26 | 4 | 98.1 | 9.8 | -4.53 | N/A | N/A | N/A | N/A |
|  | Role limitations due to emotional problems | 26 | 3 | 97.4 | 9.1 | -2.99 | N/A | N/A | N/A | N/A |
|  | Vitality | 26 | 4 | 64.0 | 18.6 | -0.36 | 0.35 | 0.84 | 0.87 | 0.92 |
|  | Mental health | 26 | 5 | 76 | 10.2 | -0.62 | 0.32 | 0.74 | 0.83 | 0.93 |
|  | Social functioning | 26 | 2 | 79.8 | 25.0 | -0.66 | 0.88 | - | 0.84 | 0.89 |
|  | (Bodily) pain | 26 | 2 | 70.7 | 26.1 | -0.32 | 0.93 | - | 0.96 | 0.96 |
|  | General health | 26 | 5 | 57.9 | 25.4 | -0.01 | 0.45 | 0.85 | 0.87 | 0.93 |
|  | Physical component score | 26 | 21 | 47.3 | 9.8 | -0.54 | 0.01 | 0.71 | 0.75 | 0.86 |
|  | Mental component score | 26 | 14 | 52.5 | 5.2 | -0.52 | 0.11 | 0.85 | 0.72 | 0.88 |

Abbreviations: α, Cronbach’s alpha; GLB, greatest lower bound; max, maximum; min, minimum; SD, standard deviation.

Table S19: Overview of the T-scores and standard errors in adults with rare bleeding disorders.

|  |  |  | **T-score** | | | | **Standard errors** | | |  |
| --- | --- | --- | --- | --- | --- | --- | --- | --- | --- | --- |
| **PROMIS item bank** | | **N** | **Mean** | **SD** | **Min** | **Max** | **Mean** | **SD** | **Min** | **Max** |
| ***Computer Adaptive Tests*** | |  |  |  |  |  |  |  |  |  |
|  | Physical function | 26 | 49.9 | 9.4 | 31.1 | 69.2 | 2.1 | 0.5 | 1.5 | 3.9 |
|  | Pain interference | 26 | 51.3 | 10.3 | 41.0 | 74.3 | 2.6 | 1.3 | 0.8 | 4.1 |
|  | Fatigue | 26 | 48.4 | 10.5 | 28.8 | 65.4 | 2.1 | 0.3 | 1.9 | 3.2 |
|  | Anxiety | 26 | 49.3 | 7.5 | 36.4 | 64.1 | 2.5 | 0.7 | 1.8 | 4.0 |
|  | Depression | 26 | 46.5 | 6.6 | 39.1 | 60.7 | 2.8 | 0.9 | 2.0 | 4.1 |
|  | Ability to participate in social roles and activities | 26 | 53.0 | 8.6 | 34.7 | 64.9 | 2.1 | 0.5 | 1.6 | 3.4 |
|  | Satisfaction with social roles and activities | 26 | 53.7 | 8.0 | 37.7 | 65.9 | 2.2 | 0.5 | 1.5 | 3.4 |
| ***Short form*** | |  |  |  |  |  |  |  |  |  |
|  | Anger | 26 | 46.3 | 7.0 | 36.3 | 58.1 | 3.3 | 0.4 | 2.8 | 4.0 |
| ***Fixed scale*** | |  |  |  |  |  |  |  |  |  |
|  | Global Health – physical health | 26 | 38.4 | 6.5 | 26.7 | 50.8 | 4.2 | 0.1 | 4.1 | 4.6 |
|  | Global Health – mental health | 26 | 32.5 | 9.3 | 21.2 | 50.8 | 3.9 | 0.4 | 3.6 | 4.6 |

Abbreviations: max, maximum; min, minimum; SD, standard deviation
